# Estrogens Promote the Production of Natural Neutralizing Antibodies in Fish through G Protein-Coupled Estrogen Receptor 1

**DOI:** 10.3389/fimmu.2017.00736

**Published:** 2017-06-29

**Authors:** María C. Rodenas, Isabel Cabas, Nuria E. Gómez-González, Marta Arizcun, José Meseguer, Victoriano Mulero, Alfonsa García-Ayala

**Affiliations:** ^1^Department of Cell Biology and Histology, Faculty of Biology, University of Murcia, IMIB-Arrixaca, Murcia, Spain; ^2^Centro Oceanográfico de Murcia, Instituto Español de Oceanografía (IEO), Murcia, Spain

**Keywords:** G protein-coupled estrogen receptor 1, estrogens, lymphocytes, natural antibodies, evolution, fish

## Abstract

Natural antibodies play crucial roles in pathogen elimination, B-cell survival and homeostasis, and inflammatory and autoimmune diseases. Although estrogens are able to regulate both innate and adaptive immune responses, their role in the production of natural antibodies is unknown. Here, we show that the dietary intake of the synthetic estradiol analog, 17α-ethinylestradiol (EE_2_), one of the most potent pharmaceutical estrogens and intensively used in human therapeutics as a component of most oral contraceptives, regulates the abundance and proliferation of T and IgM^+^ B lymphocytes in the teleost fish gilthead seabream (*Sparus aurata* L.). Furthermore, for the first time in vertebrates, it is shown that estrogen signaling through G protein-coupled estrogen receptor 1 (GPER1) induces the production of polyreactive natural antibodies, which are able to crossreact with unrelated antigens and commensal and pathogenic bacteria. In addition, the serum from fish treated with EE_2_ or the GPER1 agonist G1 shows higher complement-dependent bactericidal activity than that from non-treated specimens. These results demonstrate that estrogens and GPER1 are the key regulators of natural antibody production and pathogen clearance in fish, paving the way for future studies in other vertebrate classes.

## Introduction

The impact of estrogens on immune responses is well documented (1–4). For many years, the immune system has been considered as a natural target for estrogen action (5), as corroborated by the clear sex differences observed in autoimmune and inflammatory disorders (4).

Estrogens affect different stages of B-cell development and modify the humoral response (3), while a third generation of selective estrogen receptor (ER) modulators has been found to regulate B development and function (6). Moreover, it has recently been described that the activation of ERs directly influences antibody (Ab) expression by binding to switch sites and regulatory elements in the immunoglobulin heavy chain locus of activated B cells (7). All these effects are possible due to the presence of ER in immune cells (4), including lymphocytes (8).

Classically, the action of estrogens was thought to be mediated by nuclear ERα and ERβ, which function as hormone-inducible transcription factors by binding to the estrogen-responsive element located within the promoter region of target genes (9), although it was later confirmed that they are also able to rapidly activate transduction pathways *via* non-genomic mechanisms. These additional effects are mediated by a membrane-anchored receptor called G protein-coupled estrogen receptor 1 (GPER1), which was identified by independent laboratories in the early 2000s (10, 11) and later shown to be activated by 17β-estradiol (E_2_) (12–14). GPER1 activation downstream mechanisms include several signaling pathways involving MAPKs, ERK, PI3K, cAMP, and intracellular calcium (15, 16).

Xenoestrogens or environmental estrogens, known as endocrine disruptor chemicals (EDCs), are synthetic or natural substances of high stability that exert toxicity by mimicking the effects of estrogens (17). They have been described as being involved in autoimmunity (18, 19) and as reducing B-cell precursors in mice (20) and have also been found to activate GPER1 (21). 17α-Ethinylestradiol (EE_2_) is a synthetic analog of E_2_ and one of the most potent pharmaceutical estrogens and is intensively used in human therapeutics as a component of most oral contraceptives. The binding affinity of EE_2_ to human ERs is one to two times higher than E_2_ (22). However, it has been calculated that 16–68% of the dose is excreted in the urine or feces (23), reaching the waste water treatment plants, where cannot be totally eliminated (24). Consequently, an unquantified load of xenoestrogens are released into the aquatic environment, where they can be absorbed by sediment and persist for long periods, or taken up by animals and concentrated in their tissues (22, 25). It has been found in concentrations up to 21 ng/l in a south-western European river (Mira, Portugal) (26).

The gilthead seabream (*Sparus aurata* L.) is a seasonal marine teleost of great commercial value in the Mediterranean area. Its hermaphrodite character makes it an interesting animal model to analyze the role of estrogens, natural or synthetic, in the immune response. We have previously demonstrated that nuclear ERs and GPER1 are expressed in gilthead seabream head kidney (bone marrow equivalent) leukocytes (27, 28) and that GPER1 modulates granulocyte functions through a cAMP/protein kinase A/CREB signaling pathway (27). We also observed that EDCs altered the immune response of gilthead seabream by promoting some long-lasting effects even when their estrogenic disruptive effects were not present (29, 30). Moreover, EE_2_ bath-exposed specimens have an altered capacity to respond to an immune challenge, although the compound does not behave as an immunosuppressor (31), while the dietary intake of EE_2_ stimulates the Ab response of vaccinated fish (29, 30). However, no information exits on the impact of EE_2_ on fish lymphocyte proliferation and differentiation or, particularly, on natural antibodies. Natural antibodies are present in the serum of vertebrates without any apparent antigenic stimulation and are an important field of research for their relevance in autoimmunity and for their role as a bridge between innate and adaptive immunities (32, 33). Although the relevance of natural antibodies in fish is largely unknown, their existence in both cartilaginous and bony fish has been reported (32, 34–36). In addition, the opsonization and neutralizing ability of natural antibodies of rainbow trout against *Aeromonas salmonicida* has been demonstrated (37–39). We, therefore, examined whether estrogens, and in particular, GPER1 signaling, are able to modulate T- and B-lymphocyte responses to an immunological challenge and natural Ab production.

## Materials and Methods

### Animals, *In Vivo* Treatments, and Sample Collection

Healthy specimens of gilthead seabream were from the Oceanographic Center of Murcia (Mazarrón, Spain), where they were kept in running seawater aquaria (dissolved oxygen 6 ppm, flow rate 20% aquarium vol/h) with a natural temperature and photoperiod. They were fed three times per day with a commercial pellet diet (44% protein and 22% lipids; Skretting) at a feeding rate of 1.5% of fish biomass. The environmental parameters, mortality, and food intake, as well as behavior, were recorded daily.

**Figure 1:**
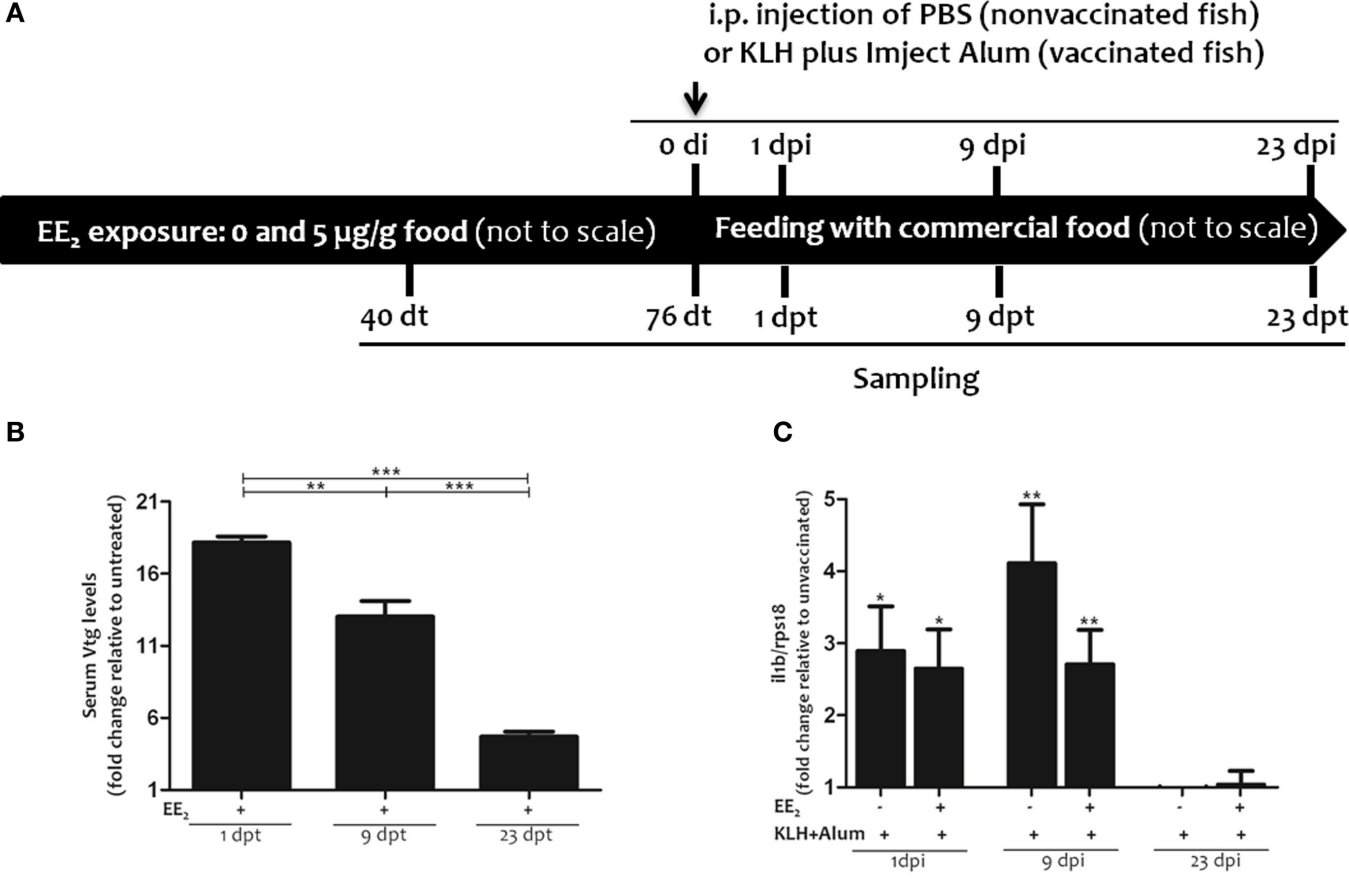
Experimental design: 17α-ethinylestradiol (EE_2_) dietary exposure and immunization protocol of gilthead seabream juveniles. **(A)**. Fish were exposed to a non-supplemented diet (untreated fish) or to a supplemented diet with 5 μg EE_2_/g food (treated fish) for 76 days (days of treatment, dt). Then, the specimens were fed with commercial food for an additional period of 23 days (days posttreatment, dpt). Fish were intraperitoneally (i.p.) injected with phosphate-buffered saline (PBS) (unvaccinated or control fish) or keyhole limpet hemocyanin (KLH) plus Imject Alum adjuvant (vaccinated fish) at 76 dt. Sampling was carried out 40 and 76 dt and 1, 9, and 23 days postinjection (dpi) (1, 9, and 23 dpt). **(B)** The levels of vitellogenin (Vtg) were determined in the serum of untreated and EE_2_ (5 μg/g food)-treated fish 1, 9, and 23 days after ceasing the treatment (dpt) by ELISA. Levels are shown as fold increase relative to the mean of untreated fish. Each bar represents mean ± SEM of duplicates of six independent fish. The sample size was *n* = 6 fish/group/time of sampling. The asterisks denote statistically significant differences after a Student’s *t*-test between the untreated and the EE_2_-treated group, at each time point and between the EE_2_-treated groups at the different time points. *p < 0.05; **p < 0.01; and ***p < 0.001. **(C)** The *il1b* mRNA levels were determined in the head kidney of untreated and EE_2_ (5 μg/g food)-treated fish (vaccinated or not) 1, 9, and 23 days postimmunization (dpi) by RT-qPCR. Gene expression levels were normalized to *rps18* mRNA levels, and data represent as the mean ± SEM of technical triplicates of six independent fish. Levels were shown as fold increase relative to the mean of non-vaccinated fish. The sample size was *n* = 6 fish/group/time of sampling. The asterisks denote statistically significant differences after a Student’s *t*-test between unvaccinated and vaccinated fish at each time point. *p < 0.05; **p < 0.01; and ***p < 0.001.

Two-month-old gilthead seabream specimens (*n* = 100/treatment), with a body weight of 26.6–63.2 (from the beginning to the end of the experiment), were exposed to EE2 (Figure 1A) in 170 l aquaria. Briefly, EE_2_ (5 μg/g food, 98% purity; Sigma) was incorporated in the commercial food using the ethanol evaporation method (0.3 l ethanol/kg of food), as described elsewhere (40). The specimens were fed three times a day *ad libitum* with the pellet diet supplemented with EE_2_ (treated fish) or non-supplemented (untreated fish) for 76 days (days of treatment, dt). Following this time, all the specimens were fed with the commercial food for a further 23 days (days posttreatment, dpt) (Figure 1A). In order to evaluate the effect of EE_2_ on the immune response, specimens were intraperitoneally (i.p.) injected with keyhole limpet hemocyanin (KLH) (45 μg/fish; Sigma-Aldrich) and Imject Alum adjuvant (4 mg/fish; Thermo Scientific) (vaccinated/immunized fish) or phosphate-buffered saline (PBS) (control/unvaccinated fish) at the end of the treatment period, 76 dt. Samples were taken 40 and 76 dt and 1, 9, and 23 days postimmunization (dpi) or dpt. Food intake was similar in all groups. Specimens (*n* = 6 fish/treatment/time of sampling) were fasted for 24 h before sampling. They were tranquilized by 8 μl/l of clove oil, immediately anesthetized using 40 μl/l of clove oil, weighed and decapitated before the head kidneys and spleens were removed, and processed for gene expression and/or flow cytometry analysis, as described later. Serum samples from trunk blood were obtained by centrifugation and immediately frozen and stored at −80°C until use. Cell suspensions from head kidney and spleen were obtained as described elsewhere (41, 42).

Another set of experiments was performed for serum Ab titer determination by ELISA: (1) 2-month-old gilthead seabream specimens were dietary treated with EE_2_ (5 μg/g food) or the GPER1 agonist G1 (5 μg/g food; Sigma-Aldrich) for 110 days (30), (2) adult specimens (650 g mean weight) were treated with 0, 5, and 50 ng/l EE_2_ for 2 months by bath immersion (31), and (3) adult specimens (225 g mean weight) were exposed to dietary G1 (0, 2, and 20 μg/fish/day treatment) for 50 days (27).

### Determination of Serum Vitellogenin (Vtg) Levels

The serum Vtg levels were determined by the enzyme-linked immunosorbent assay using a commercial kit (Cayman Chemical), following the manufacturer’s instructions. In brief, an aliquot of 1:500 diluted serum from untreated and EE_2_-treated fish (*n* = 6 fish/treatment/time of sampling) was added to flat-bottomed 96-well plates, followed by a commercial polyclonal Ab against gilthead seabream Vtg (1:100) and an antirabbit IgG (whole molecule)-peroxidase Ab (1:1,000) (Sigma-Aldrich). Finally, the chromogen tetramethylbenzidine (TMB) was added and the absorbance was read at 450 nm using an FLUOstart luminometer (BGM; LabTechnologies).

### Analysis of Gene Expression

Total RNA was extracted from head kidney from untreated and EE_2_-treated (vaccinated or not) fish (*n* = 6 fish/treatment/time of sampling) at 1, 9, and 23 dpi/dpt with TRIzol Reagent (Thermo Fisher Scientific), following the manufacturer’s instructions, and quantified with a spectrophotometer (NanoDrop, ND-1000). The RNA was then treated with amplification grade DNase I (1 U/μg RNA; Thermo Fisher Scientific) to remove genomic DNA traces that might interfere with the PCRs, and the SuperScript III RNase H Reverse Transcriptase (Thermo Fisher Scientific) was used to synthesize first-strand cDNA with oligo-dT18 primer from 1 mg of total RNA for 50 min at 50°C. The β-actin (*actb*) gene was analyzed by PCR performed with an Eppendorf Mastercycle Gradient Instrument (Eppendorf) to check cDNA quality. Reaction mixtures were incubated for 2 min at 94°C, followed by 30 cycles of 45 s at 94°C, 45 s at the specific annealing temperature (55°C), 45 s at 72°C, and finally, 10 min at 72°C.

In the same samples, the expression levels of the gene coding for the proinflammatory cytokine interleukin-1β (*il1b*) were analyzed by real-time PCR performed with an ABI PRISM 7500 instrument (Applied Biosystems) using the SYBR Green PCR Core Reagents (Applied Biosystems). Reaction mixtures were incubated for 10 min at 95°C, followed by 40 cycles of 15 s at 95°C, 1 min at 60°C, and finally, 15 s at 95°C, 1 min at 60°C, and 15 s at 95°C. The gene expression was corrected by the ribosomal protein S18 gene (*rps18*) content in each sample using the comparative cycle threshold method, Ct method (2^−ΔΔCt^). The gilthead seabream-specific primers used are listed in Table 1. In all cases, samples were analyzed in triplicate.

**Table 1:** Gene accession numbers and primer sequences used for expression analysis.

Gene	Accession no.	Name	Sequence (5′/30)
*actb*	X89920	F3	ATCGTGGGGCGCCCCAGGCACC
		R3	CTCCTTAATGTCACGCACGATTTC
*il1b*	AJ277166	F2	GGGCTGAACAACAGCACTCTC
		R3	TTAACACTCTCCACCCTCCA
*rps18*	AM490061	F	AGGGTGTTGGCAGACGTTAC
		R	CTTCTGCCTGTTGAGGAACC

*The gene symbols followed the Zebrafish Nomenclature Guidelines (http://zfin.org/zf_info/nomen.html). All primers were used for real-time PCR, except *actb* primers that were used for conventional PCR*.

### Immunofluorescence and Flow Cytometry

The percentage of Zap70-, IgM-, and Pax5-positive cells was determined by flow cytometry (Figure S1A in Supplementary Material). In brief, cytoplasmic Zap70 and Pax5, and surface and total IgM, were detected in aliquots of 0.5 × 10^6^ head kidney leukocytes at 1, 9, and 23 dpi/dpt and of spleen leukocytes at 23 dpi/dpt (*n* = 6 fish/treatment/time of sampling) from untreated and EE_2_-treated fish (vaccinated or not). The leukocytes were washed with PBS containing 2% fetal calf serum (FCS; Thermo Fisher Scientific) and 0.05% sodium azide (FACS buffer). Cells were fixed with 4% paraformaldehyde for 15 min at room temperature. After three rinses, cells were incubated in ice-cold PBS containing 1% BSA and saponin (Thermo Fisher Scientific) at 4°C to permeabilize the plasma membrane. Cells were then stained with 0 and 2 μg/ml (0, 1:100) of the 99F2 rabbit monoclonal Ab to Zap70 (Cell Signaling) (43) or mouse monoclonal Ab specific to seabream IgM (Aquatic Diagnostics) (44), in PBS containing 2% FCS for 30 min at 4°C. After washing, cells were incubated with a 1:1,000 dilution of a phycoerythrin-conjugated antirabbit or -mouse Ig Ab, respectively, for 30 min at 4°C, and washed again twice. In other experiments, cells were also stained with 2 μg/ml (1:100) of the D19F8 rabbit monoclonal Ab to Pax5 conjugated with Alexa 488 (Cell Signaling) (43) in PBS containing 2% FCS for 1 h at room temperature. After washing, cells were analyzed in a flow cytometer (BD Biosciences).

### Proliferation Assay

To assess the proliferative activity of head kidney and spleen Zap70^+^ and IgM^+^ cells at 1, 9, and 23 dpi/dpt (*n* = 6 fish/treatment/time of sampling), 5-ethynyl-2′-deoxyuridine (EdU) (Thermo Fisher Scientific) was i.p. injected 2 h before sampling. Then, aliquots of 0.5 × 10^6^ head kidney and spleen cells were used to determine the percentage of double positive cells, i.e., Zap70^+^/EdU^+^ and IgM^+^/EdU^+^. EdU^+^ cells were detected by fluorescent-azide coupling reaction with EdU according to the manufacturer’s protocol (Click-iT; Thermo Fisher Scientific). Zap70^+^ and IgM^+^ were labeled as described earlier and analyzed by flow cytometry (Figure S1 in Supplementary Material). The percentage of positive cells is given on head kidney R2 region (FSC^low^/SSC^low^), which includes macrophages, lymphocytes, and hematopoietic precursor cells and excludes acidophilic granulocytes (R1: FSC^high^/SSC^high^) (44).

### Determination of Serum IgM Titer

Total IgM titers and those specific to KLH, lysozyme (unrelated antigen), *Shewanella putrefaciens* strain Pdp11 (a skin microbiota bacterium from gilthead seabream) (45), or *Vibrio anguillarum* strain R82 (a fish pathogen) were determined in serum by ELISA (Aquatic Diagnostics), following the manufacturer’s instructions. In brief, serial dilutions of pooled serum samples (*n* = 6 fish/treatment/time of sampling) were added to flat-bottomed 96-well plate precoated with KLH (1 μg/well), chicken egg white lysozyme (1 μg/well; Sigma-Aldrich), and *S. putrefaciens* or *V. anguillarum* (10^6^ bacteria/well), followed by a monoclonal Ab specific to gilthead seabream IgM (1:100) and then an antimouse IgG peroxidase Ab (1:1,000) (Sigma-Aldrich). Finally, the chromogen TMB was added and the absorbance was read at 450 nm using an FLUOstart luminometer (BGM; LabTechnologies). As negative controls, the serum or the primary Ab was omitted.

### Bactericidal Activity

*Vibrio anguillarum* strain R82 was grown in tryptic soy agar plates at 25°C. Fresh single colonies were diluted in 5 ml of tryptic soy broth, cultured for 16 h at 25°C on an orbital incubator at 300 rpm, and adjusted to 10^7^ bacteria/ml. Aliquots of 50 μl containing 10^6^ bacteria in HBSS with Ca^2+^/Mg^2+^ were placed in flat-bottomed 96-well plates and incubated for 2 h with 50 μl of pool gilthead seabream serum samples diluted 1/10 in PBS. Moreover, several controls were introduced: blank, without bacteria; positive control, with 10 μg/ml gentamicin (0% growth or 100% bactericidal activity); negative control, without serum (100% growth or 0% bactericidal activity); and decomplemented serum control (pretreated at 50°C, 20 min) to asses complement-mediated killing. The bactericidal activity of serum samples was determined using a 2,3-bis(2-methoxy-4-nitro-5-sulfophenyl)-2*H*-tetrazolium-5-carboxanilide inner salt (XTT) assay where the yellow tetrazolium salt XTT is reduced to a colored formazan dye by dehydrogenase enzymes in metabolically active cells (46). The bactericidal activity was expressed as the percentage of bacterial growth inhibition.

### Statistical Analysis

Data were analyzed by the analysis of variance. An unpaired Student’s *t*-test was applied to determine differences between two groups. The critical value for statistical significance was taken as *p* < 0.05. The asterisks *, **, and *** refer to *p* < 0.05, *p* < 0.01, and *p* < 0.001, respectively. All statistical analyses were carried out using the GraphPad Prism 5 program.

## Results

### EE_2_ Increases Vtg Serum Levels But Hardly Affects *il1b* Expression in Head Kidney

The survival of gilthead seabream specimens was 100% during the trial (data not shown). As a control for estrogenic endocrine disruption, serum Vtg levels were analyzed. The results showed that the dietary intake of EE_2_ significantly increased serum Vtg levels, as we have previously described for hepatic *Vtg* transcript levels in adult and juvenile gilthead seabream fish treated with EE_2_ (29–31, 47, 48). Vtg levels peaked at the end of the treatment time and then gradually decreased, although they were still altered at 23 dpt (Figure 1B). Furthermore, when the expression of the gene coding for *il1b* was analyzed in head kidney at 1, 9, and 23 dpi to assess the activation of innate immunity in response to the immunization, increased *il1b* transcript levels were only evident 1 and 9 dpi as expected (Figure 1C). However, dietary EE_2_ failed to significantly affect *il1b* mRNA levels (Figure 1C).

### EE_2_ Affects the Abundance and Proliferation of T Lymphocytes

**Figure 2:**
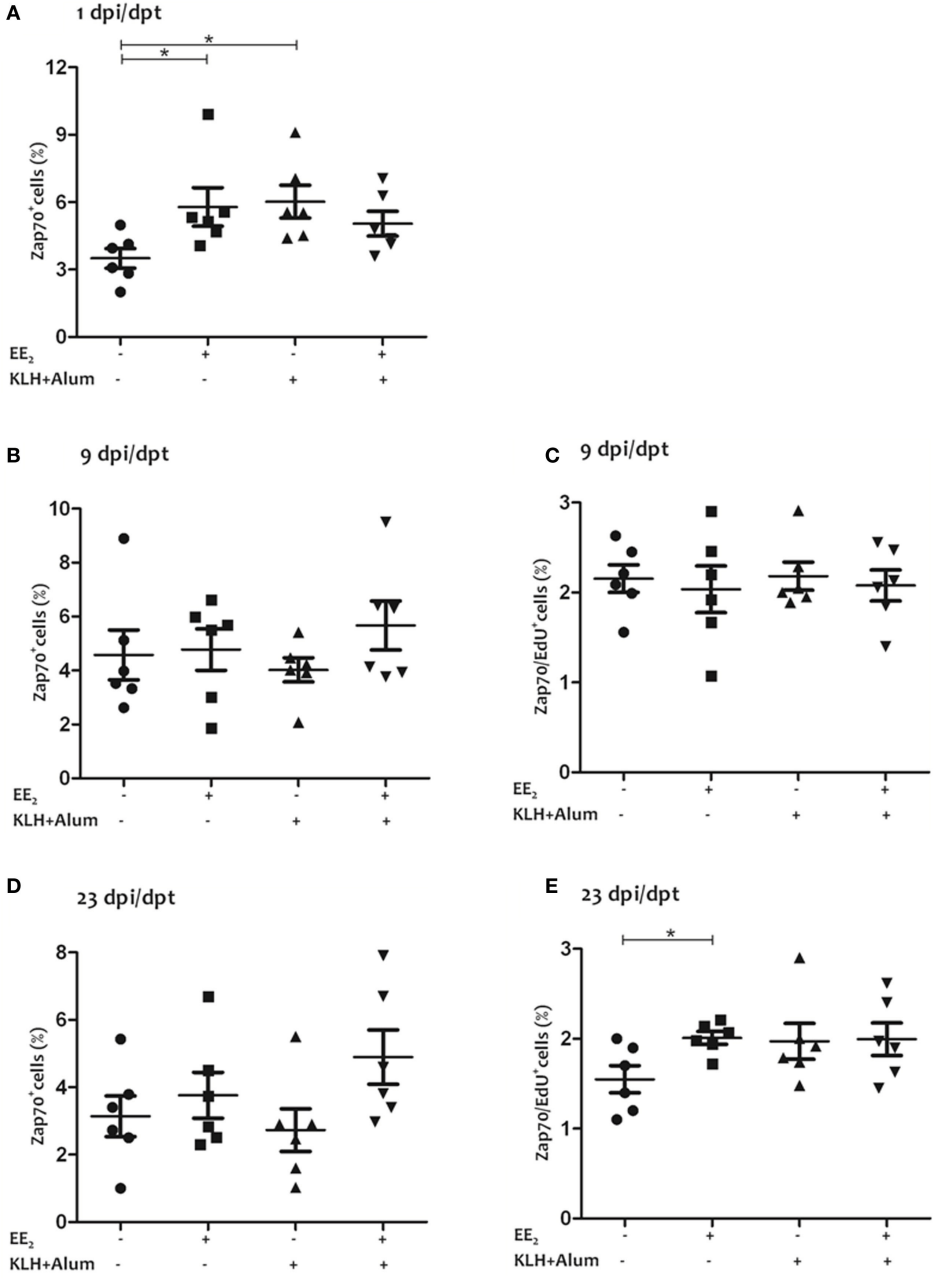
17α-ethinylestradiol (EE_2_) exposure modulates the abundance and proliferation of T lymphocytes. The percentage of Zap70^+^ cells **(A,B,D)** and that of proliferating Zap70^+^ cells (i.e., EdU^+^ cells) **(C,E)** were determined in head kidney leukocytes from untreated and EE_2_ (5 μg/g food)-treated fish (vaccinated or not) 1 **(A)**, 9 **(B,C)**, and 23 **(D,E)** days after injection and posttreatment (dpi, dpt) by flow cytometry. The sample size was *n* = 6 fish/group/time of sampling. The mean for each group of specimens is shown as a horizontal line. The percentage is given on head kidney R2 region, which contains macrophages, lymphocytes, and precursor cells, excluding acidophilic granulocytes (49). The asterisks denote statistically significant differences after a Student’s *t*-test between the indicated groups. *p < 0.05.

Both the dietary intake of EE_2_ and immunization led to an increase in the percentage of T lymphocytes (i.e., Zap70^+^ cells) in head kidney at 1 dpi (Figure 2A), whereas no statistically significant differences were observed at 9 and 23 dpi with any of the treatments (Figures 2B,D). Although the percentage of proliferating T lymphocytes (i.e., Zap70^+^/Edu^+^ cells) in head kidney was unaffected by the treatments at 9 dpi (Figure 2C), EE_2_ slightly increased the percentage of proliferating T lymphocytes in the head kidney at 23 dpi (Figure 2E).

### EE_2_ Alters IgM^+^ B-Lymphocyte Populations

**Figure 3:**
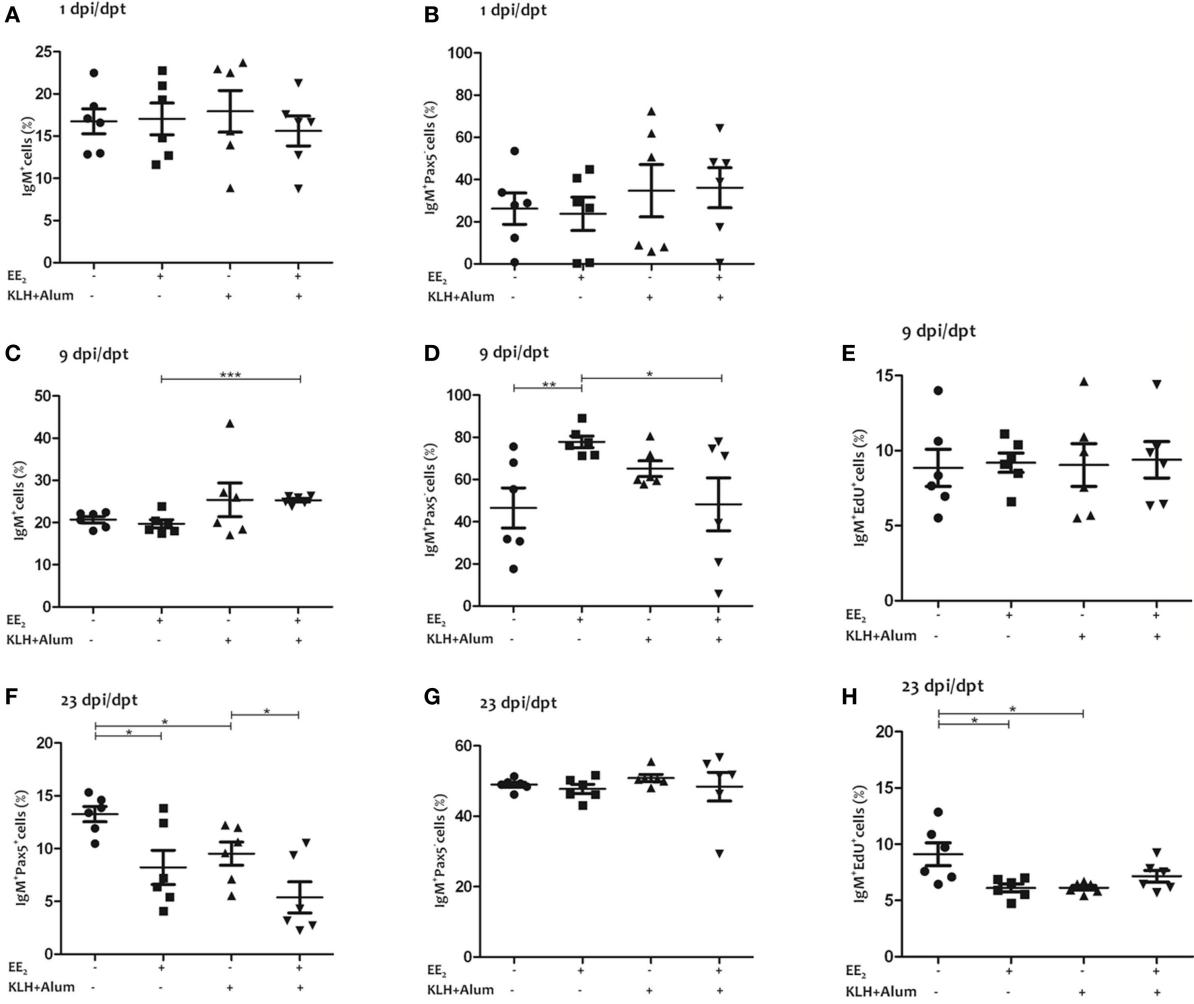
17α-Ethinylestradiol (EE_2_) exposure promotes the differentiation of IgM^+^ B lymphocytes. The percentage of total IgM^+^ cells (surface and intracellular staining) in the head kidney **(A,C,F)** and the percentage of IgM^+^/Pax5^−^ cells **(B,D,G)**, and IgM^+^/EdU^+^ cells **(E,H)** of total IgM^+^ cells were determined in head kidney leukocytes from untreated and EE_2_ (5 μg/g food)-treated fish (vaccinated or not) 1 **(A,C,F)**, 9 **(B,D,G)**, and 23 **(E,H)** dpi (1, 9, and 23 dpt) by flow cytometry. The sample size was *n* = 6 fish/group/time of sampling. The mean for each group of specimens is shown as a horizontal line. The percentage is given on head kidney R2 region, which contains macrophages, lymphocytes, and precursor cells, excluding acidophilic granulocytes (49). The asterisks denote statistically significant differences after a Student’s *t*-test between the indicated groups. *p < 0.05, **p < 0.01 and ***p < 0.001.

Although the percentage of IgM^+^ B lymphocytes in the head kidney was slightly higher in EE_2_/immunized fish than in the control at 9 dpi/dpt, it was lower in all treated group than in the control at 23 dpi/dpt (Figures 3A,C,F). Interestingly, although the percentage of IgM^+^/Pax5^−^ B lymphocytes, i.e., plasma cells, was unaltered at 1 and 23 dpi/dpt, it was higher at 9 dpi/dpt in EE_2_/non-immunized fish but not in immunized fish treated with EE_2_ (Figures 3B,D,G). In addition, the dietary intake of EE_2_ and immunization resulted in a decreased proliferation of IgM^+^ B lymphocytes at 23 dpi/dpt (Figures 3E,H). Neither immunization nor EE_2_ exposure was seen to modulate IgM^+^ B-lymphocyte abundance, proliferation, or differentiation in the spleen at 23 dpi/dpt (data not shown).

### EE_2_ Induces the Production of Natural Neutralizing Antibodies

**Figure 4:**
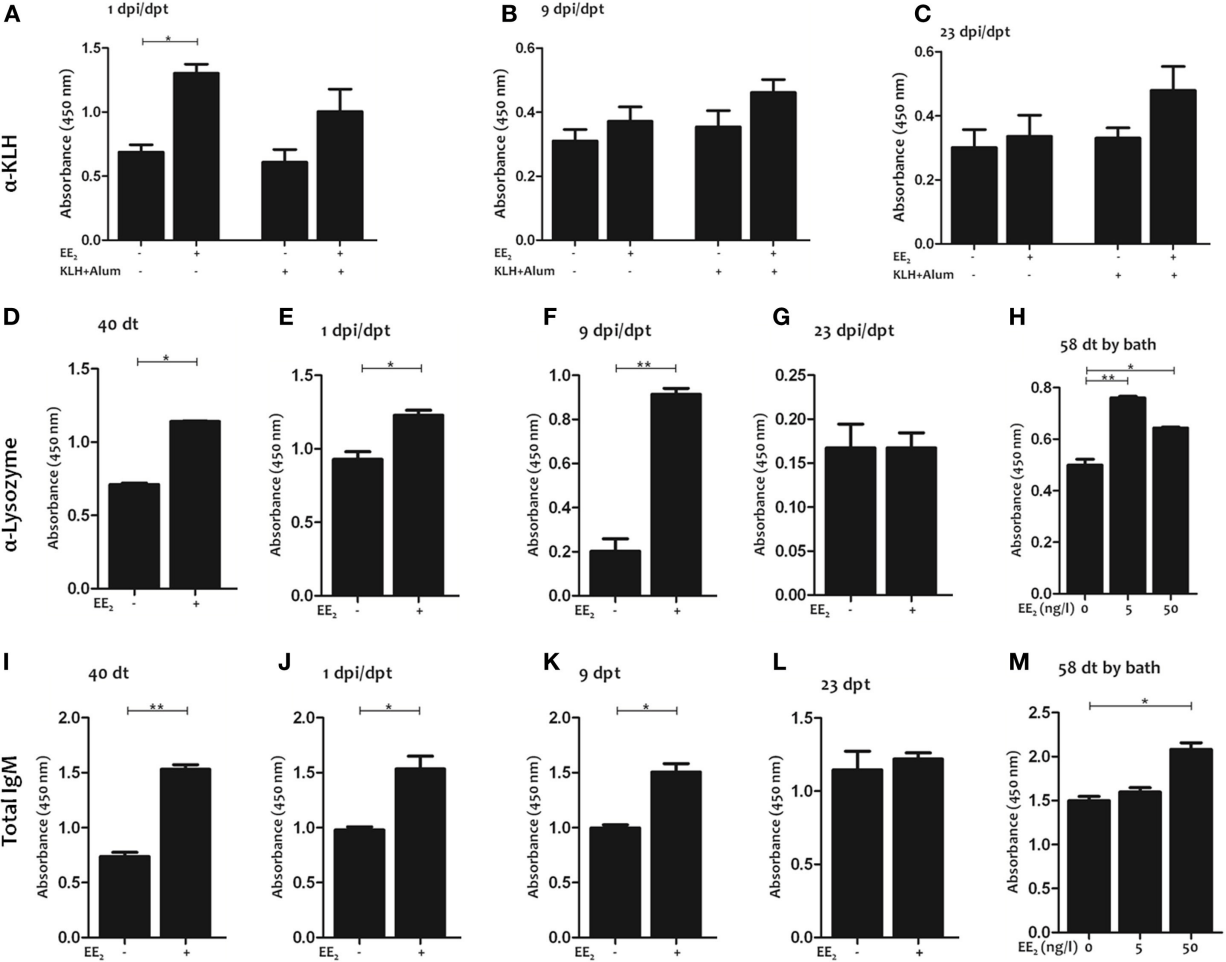
17α-Ethinylestradiol (EE_2_) exposure induces the production of natural antibodies. Keyhole limpet hemocyanin (KLH)-specific **(A–C)**, lysozyme-specific **(D–G)**, and total **(I–L)** IgM titers were determined in the serum by ELISA 1 **(A,E,J)**, 9 **(B,F,K)**, and 23 **(C,G,L)** dpi (1, 9, and 23 dpt, respectively) and 40 dt **(D,I)** in untreated and EE_2_ (5 μg/g food)-treated fish (vaccinated or not). In addition, lysozyme-specific **(H)** and total **(M)** IgM titers were determined in the serum of specimens bath exposure for 58 days to 0, 5, and 50 ng/l EE^2^. The data represent the mean ± SEM of absorbance value of the pooled sera of six individual fish. The sample size was = 6 fish/group/time of sampling. The asterisks denote statistically significant differences after Student’s *t*-test between the untreated and EE_2_-treated fish (vaccinated or not), at each time point. *p < 0.05 and **p < 0.01.

First, the KLH-specific IgM titer was analyzed by ELISA in serum from untreated and EE_2_-treated fish, both control and immunized, at 1, 9, and 23 dpi/dpt. Unexpectedly, the dietary intake of EE_2_ significantly increased the KLH-specific IgM titer at 1 dpi/dpt (Figure 4A). However, immunization failed to elicit a specific IgM response at any of the times analyzed (Figures 4A–C), presumably reflecting the poor primary Ab response to KLH.

**Figure 5:**
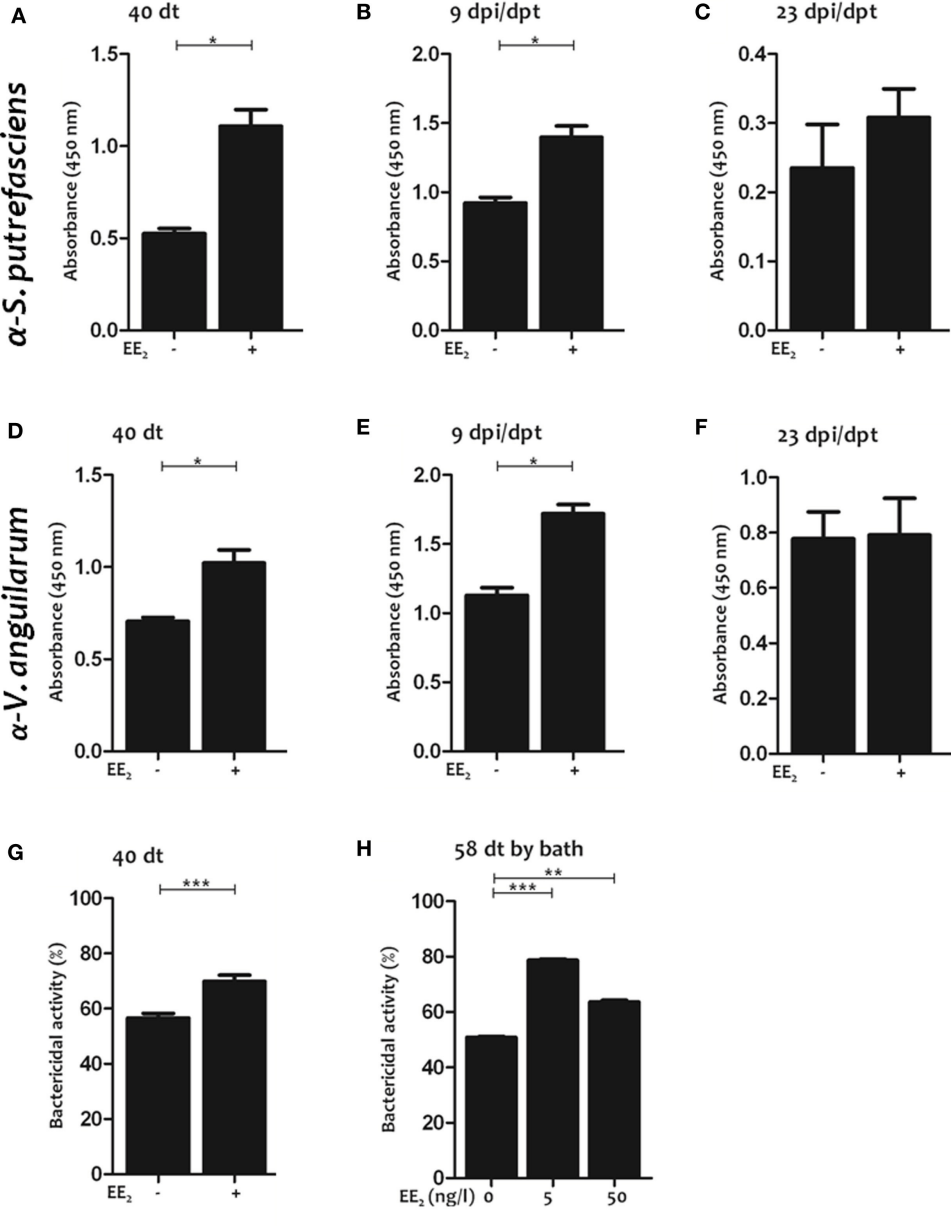
17α-Ethinylestradiol (EE_2_)-induced natural antibodies react against commensals and neutralize pathogens. **(A–F)** The natural IgM titers against *Shewanella putrefaciens* or *Vibrio anguillarum* were determined in the serum by ELISA after 40 days of treatment **(A,D)** and after 9 **(B,E)** and 23 **(C,F)** dpt in untreated and EE_2_ (5 μg/g food)-treated fish. The data represent the mean ± SEM of absorbance value of pool sera of six individual fish at 1:10 serum dilution. **(G,H)** The bactericidal activity against *V. anguillarum* of the serum of untreated and EE_2_ (5 μg/g food)-treated fish at 40 dt **(G)** and EE_2_ (0, 5, and 50 ng/l) bath exposure adult fish for 58 days **(H)** was determined by an XTT assay. The data represent the mean ± SEM of percentage of bacterial activity by pool sera of six individual fish at 1:10 serum dilution. The simple size was = 6 fish/group/time of sampling. The asterisks denote statistically significant differences after Student’s *t*-test between the untreated and EE_2_-treated fish, at each time point. *p < 0.05; **p < 0.01; and ***p < 0.001.

**Figure 6:**
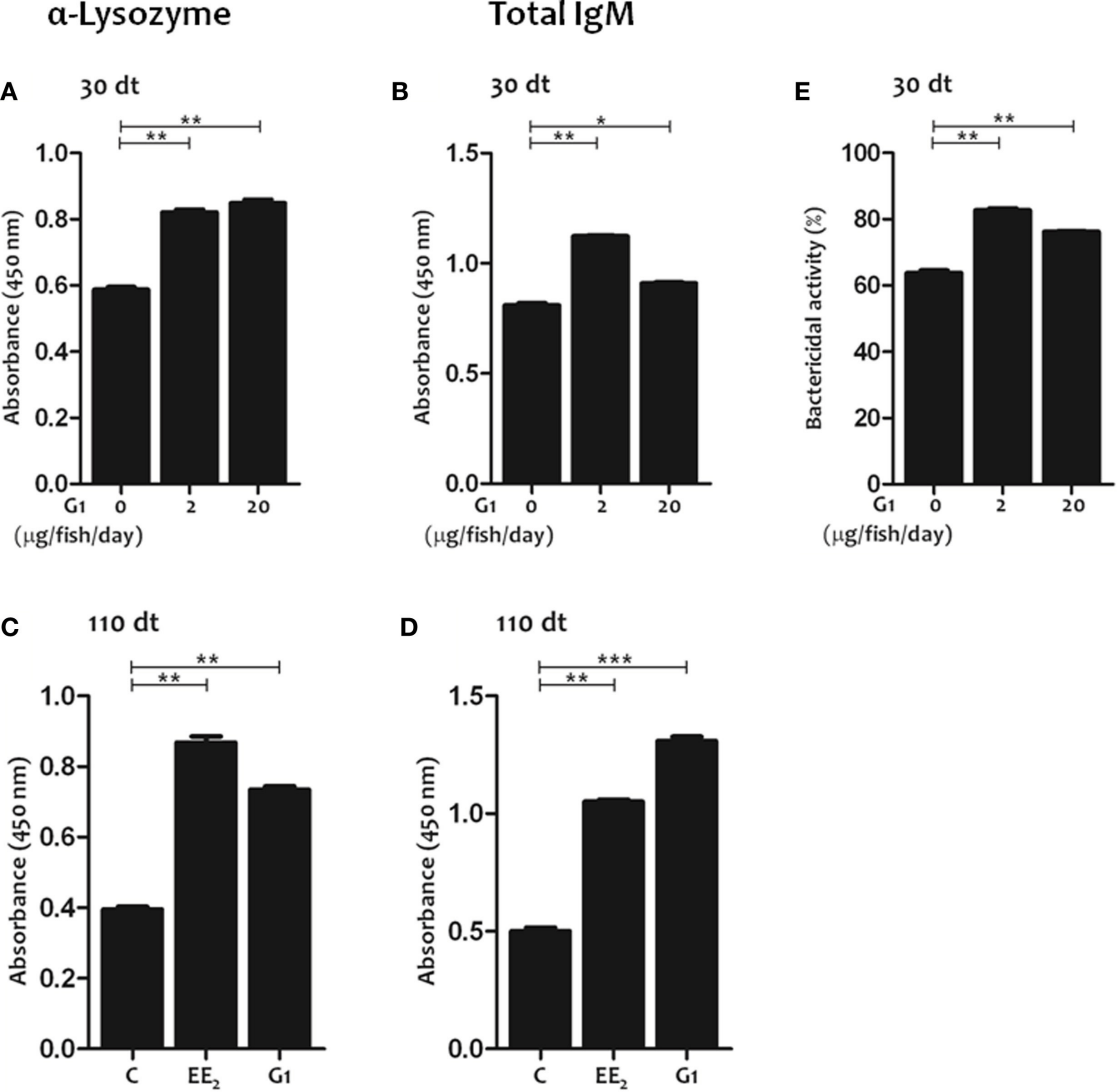
G protein-coupled estrogen receptor 1 activation *in vivo* promotes the production of natural antibodies. **(A–D)** Lysozyme-specific **(A,C)** and total **(B,D)** IgM titers were determined in the serum by ELISA after 30 and 110 days of dietary intake of G1 (0, 2, and 20 μg/fish/day) **(A,B,E)**, or 17α-Ethinylestradiol (EE_2_) (5 μg/g food) and G1 (5 μg/g food) **(C,D)**. The data represent the mean ± SEM of absorbance value of the pooled sera from six individual fish at 1:10 serum dilution. The sample size was = 6 fish/group/time of sampling. **(E)** The serum bactericidal activity against *Vibrio anguillarum* of fish exposure for 30 days to G1 (0, 2, and 20 μg/fish/day) was determined by an XTT assay. The data represent the mean ± SEM of percentage of bacterial activity by pool sera of six individual fish at 1:10 serum dilution. The sample size was = 6 fish/group/time of sampling. The asterisks denote statistically significant differences after Student’s *t*-test between the untreated and EE_2_-treated fish, at each time point. *p < 0.05; **p < 0.01; and ***p < 0.001.

These results prompted us to analyze the IgM titer to an unrelated antigen, lysozyme, in the serum of non-immunized fish exposed to EE_2_ by different routes (in this case dietary exposure and bath immersion). Strikingly, EE_2_ significantly increased lysozyme-specific IgM titers during treatment (40 dt) (Figure 4D) and posttreatment (1 and 9 dpt) (Figures 4E,F), but the effect did not last until 23 dpt (Figure 4G). Similar results were obtained in fish bath-exposed to EE_2_ for approximately 2 months (Figure 4H). The induction of natural antibodies by the administration of EE_2_ was further confirmed by determining total serum IgM levels (Figures 4I–M). Furthermore, EE_2_-induced natural IgM antibodies were not only polyreactive but also neutralizing, since they reacted against the commensal *S. putrefaciens* (Figures 5A–C) and the pathogen *V. anguillarum* (Figures 5D–F), inducing the death of the latter (Figures 5G,H). In all cases, the natural antibodies show low affinity, since high dilution of serum had to be used to detect them (Figures 4–6).

### GPER1 Signaling Promotes Natural Ab Production

As previous studies demonstrated the key role of estrogen signaling through GPER1 in the regulation of innate and adaptive immunities in gilthead seabream, we next evaluated the effect of GPER1 activation on natural Ab production using the specific agonist G1. The results showed that G1-treated fish had higher lysozyme-specific (Figure 6A) and total IgM (Figure 6B) serum titers and higher bactericidal activity against *V. anguillarum* at 30 dt than the untreated controls (Figure 6E). Moreover, the effects of EE_2_ and G1 on natural Ab production in fish treated for 110 days were similar (Figures 6C,D).

## Discussion

The fact that estrogens have a key as modulator role of mammals and fish immune systems is well known (4, 29–31, 47). However, the effect of estrogens on fish lymphocytes, and in particular, on the production of natural antibodies, is unknown. In this study, we report for the first time the capacity of EE_2_ to modulate lymphocyte populations and natural Ab production in teleost fish. We have seen that the dietary intake of EE_2_ increased the abundance and proliferation of T lymphocytes in the head kidney of unvaccinated gilthead seabream specimens. In contrast, a reduction in the number of circulating lymphocytes was observed in fathead minnow exposed to potent estrogenic effluents (50), whereas E_2_ bath exposure altered thymus development in European seabass (51). Unfortunately, the functional consequences of these observations were not investigated.

As regards, IgM^+^ B lymphocytes, the dietary intake of EE_2_, did not affect their abundance in the two main lymphomyeloid organs, namely the head kidney and the spleen, confirming previous results (29, 30). In contrast, another EDC, tamoxifen, was able to increase the number of IgM^+^ B lymphocytes in vaccinated fish (29, 30), suggesting that each EDC may have specific effects in fish adaptive immunity. Thus, it was observed in the present study that dietary EE_2_ exposure transiently increased the abundance of plasma cells, i.e., IgM^+^/Pax5^−^ cells. This suggest that EE_2_ is able to modulate B-cell lineage commitment, as Pax5 is (i) expressed from the pro-B cell through mature and activated B-cell stages, (ii) downregulated during terminal differentiation, and (iii) absent at the plasma cell stage in both mammals (52) and fish (53).

The most interesting observation in this study is that EE_2_, following dietary or bath exposure, induces the production of low affinity, polyreactive natural antibodies. Although we have previously described several long-lasting effects of dietary EE_2_ in specific Ab production (29, 30), the modulation of natural Ab production by dietary EE_2_ disappears by 23 dpt. Therefore, the stimulation of natural antibodies by EE_2_ coincides with its effect on IgM^+^ B-lymphocyte differentiation. Moreover, it was observed that the sera from EE_2_-treated fish manifested an enhanced bactericidal activity, suggesting that natural antibodies are able to effectively neutralize fish pathogens. In mammals, the primary roles of natural antibodies are to confine infection locally, to enhance the IgG response, to play a protective role in autoimmunity, and to take part in homeostasis and clearing of cell debris (54). Natural antibodies are mostly IgM type and are secreted by the long-lived, self-renewing B1 subset of B cells, which are generated during fetal or neonatal development (55, 56). Teleost B cells resemble mammalian B1 cells since both of them show phagocytic and microbicidal activities (57). Although the relevance of natural antibodies in fish is largely unknown, their existence in both cartilaginous and bony fish has been reported (34–36). More recently, the existence of natural Ab in cod has also been shown even though this species is a poor Ab responder (32) and lacks CD4 and MHCII (58). In addition, the opsonization and neutralizing ability of natural antibodies of rainbow trout against *A. salmonicida* has been demonstrated (37–39).

Although estrogens regulate Ab production, including the production of autoantibodies (19, 59, 60), their impact on natural Ab production is largely unknown. It has been reported that chronic estrogen administration (31 weeks) to mice resulted in increased IgM- and IgG-producing cells (61). However, the mechanisms involved and whether this effect results in increased natural antibodies titers are unknown. Therefore, to the best of our knowledge, this is the first study showing that estrogens regulate natural Ab production in vertebrates. In addition, this effect is mediated through GPER1 signaling, since the GPER1-specific agonist induces a similar natural Ab response to EE_2_. Our results pave the way for future studies aimed at shedding light on the relevance of estrogens in the protection of fish by natural antibodies, the signaling mechanism involved, including classical nuclear ERs, the impact of natural antibodies and estrogens in the crosstalk between natural antibodies, both IgM and IgT, and microbiota, and the therapeutic potential of estrogen administration to protect fish against infections in aquaculture.

## Ethics Statement

The experiments described were approved by the Consejería de Agua, Agricultura y Medio Ambiente of the Región de Murcia, Spain (approval number A13160507).

## Author Contributions

AG-A and VM conceived and designed the study; MR performed the research; MR, IC, NG-G, MA, JM, AG-A, and VM analyzed the data; and MR, IC, and VM wrote the manuscript with minor contribution from other authors.

## Conflict of Interest Statement

The authors declare that the research was conducted in the absence of any commercial or financial relationships that could be construed as a potential conflict of interest.
