# Supplementary figures and images for: Estrogens Promote the Production of Natural Neutralizing Antibodies in Fish through G Protein-Coupled Estrogen Receptor 1
Source: Front Immunol. 2017 Jun 29;8:736. doi: 10.3389/fimmu.2017.00736 (PMC5489559; doi:10.3389/fimmu.2017.00736)

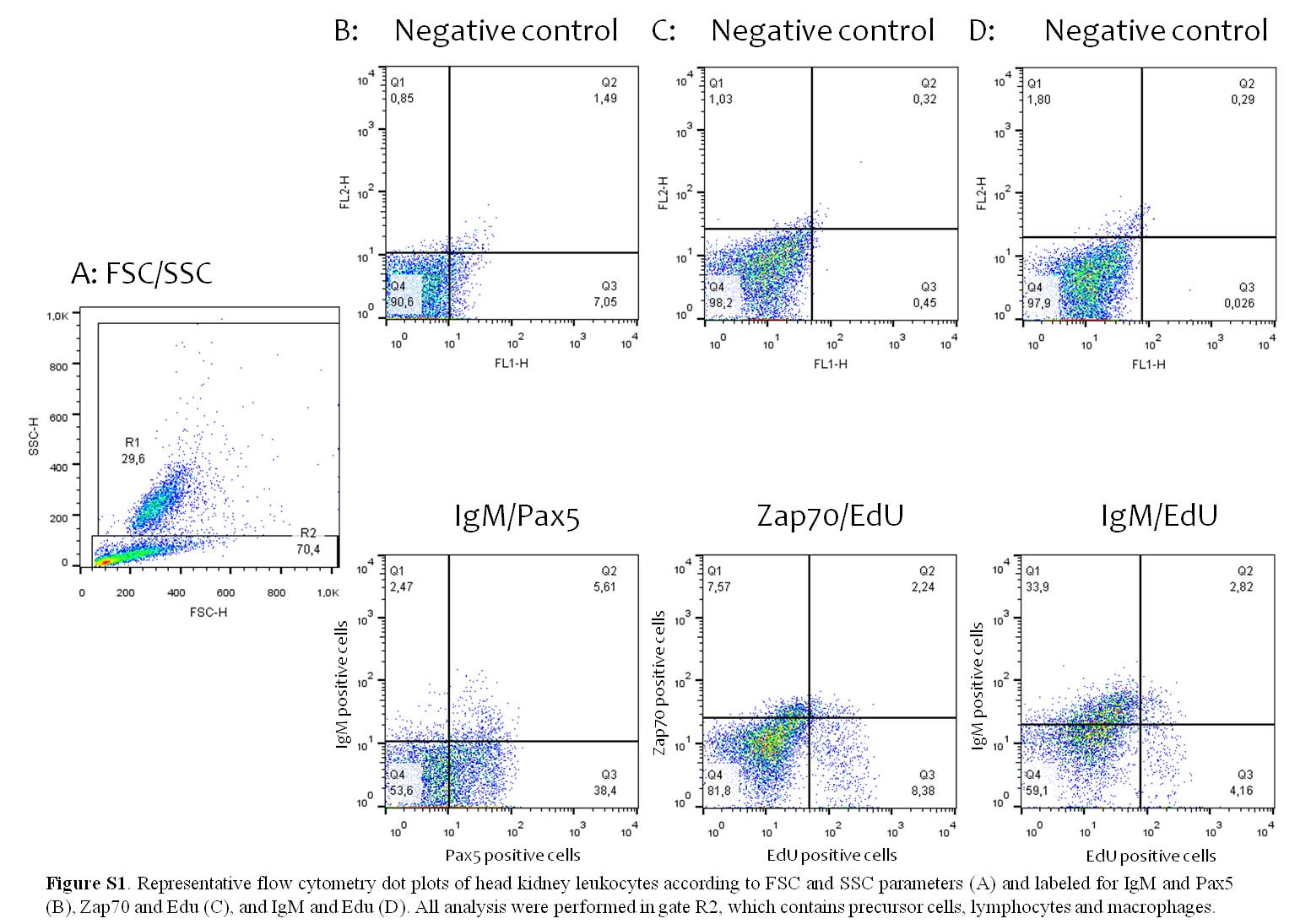

Supplement: Supplementary file 1 [file Image_1.JPEG]
